# Supplementary material for: Contact-independent killing mediated by a T6SS effector with intrinsic cell-entry properties
Source: Nat Commun. 2021 Jan 18;12:423. doi: 10.1038/s41467-020-20726-8 (PMC7813860; doi:10.1038/s41467-020-20726-8)
Supplement: Supplementary file 1 — Supplementary information [file 41467_2020_20726_MOESM1_ESM.pdf]

Supplementary Information

Contact-independent killing mediated by a T6SS effector with intrinsic cell-entry properties

This PDF file includes:

Supplementary Figures 1-14

Supplementary Tables 1-2

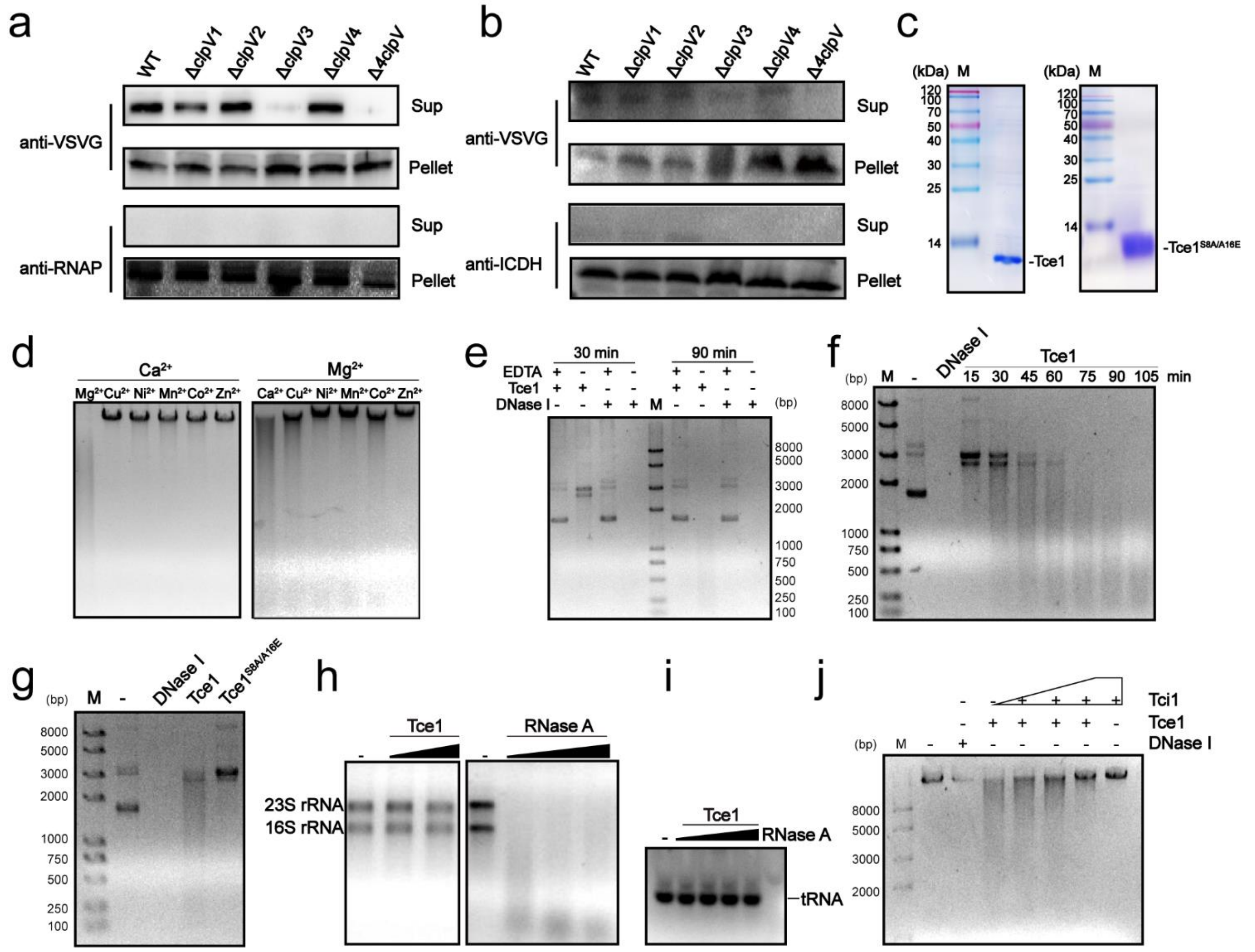

Supplementary Figure 1: YPK_0954 (Tce1) is a T6SS-3 secreted nuclease effector and YPK_0955 (Tci1) is its immunity protein. a-b, YPK_0952 and YPK_0954 are T6SS-3 effectors. Plasmids directing the expression of YPK_0952-VSVG (**a**) or YPK_0954-VSVG (**b**) were introduced into the indicated *Y. pseudotuberculosis* strains. Total cell pellet (Pellet) and secreted proteins in culture supernatant (Sup) were isolated and probed for the presence of the fusion protein. The cytosolic RNA polymerase (RNAP) or isocitrate dehydrogenase (ICDH) was similarly detected as a control. **c,** His_6-Tce1 and His_6-Tce1$^{S8A/A16E}$ proteins expressed in *E. coli* were purified to homogeneity using chromatography and analyzed with SDS-PAGE. **d,** Divalent metal depending DNase activity assay of Tce1. Tce1 was incubated with λ DNA (0.35 μg) in the presence of Ca^{2+} with other divalent metal ions (Left), or Mg^{2+} with other divalent metal ions (Right). The products were analyzed using agarose gel. **e,** DNase activity assay of Tce1 with plasmid DNA as the substrate. Tce1 was incubated with pUC19 plasmid DNA in the same reaction buffer as DNase I with or without EDTA at 37°C for 30 and 90 min. **f,** The time course of pUC19 cleavage by Tce1. Tce1 was incubated with pUC19 DNA at 37°C for 15 to 105 min. **g,** The DNase activity assay of Tce1 and Tce1$^{S8A/A16E}$ with pUC19 DNA as the substrate. **h-i,** The rRNA (**h**) and tRNA (**i**) hydrolysis activity assay of Tce1. Various amounts of Tce1 or RNase A were incubated with rRNA (**h**), or tRNA (**i**) at 37°C for 30 min, and analyzed by agarose gel electrophoresis. **j,** Tci1 inhibited the DNase activity of

Tce1. Purified Tce1 protein (0.009 μM) was incubated with λ DNA (0.35 μg) in the absence or presence of Tci1 (0, 0.005, 0.010 and 0.015 μM) in the DNase reaction buffer for 30 min at 37°C. Reaction products were analyzed through agarose gel electrophoresis. All these gels and blots were obtained from three independent experiments.

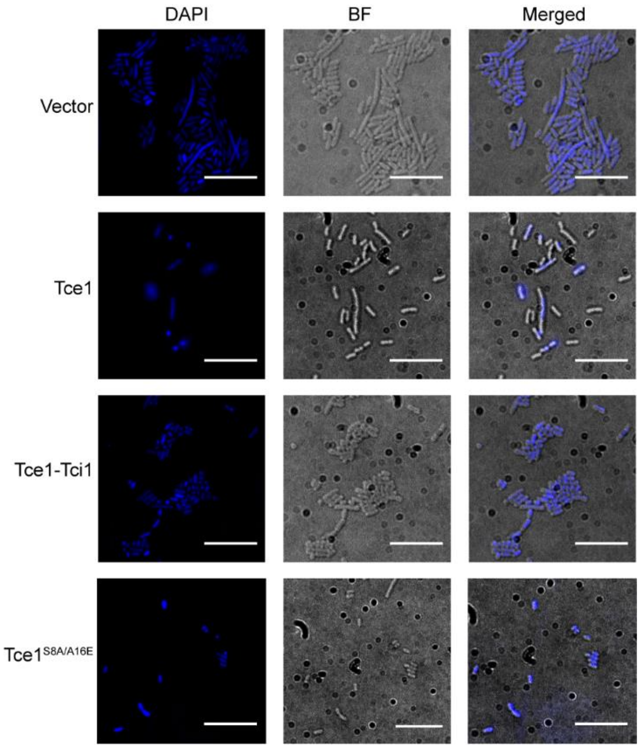

Supplementary Figure 2: Tce1 functions as DNase *in vivo*. Tce1 activity was detected by observing the loss of DNA staining (DAPI) in indicated *E. coli* cells 4 h after IPTG induction with fluorescence microscopy. Separate channels of induced *E. coli* BL21(DE3) with pET28a, pET28a-*tce1*, pET28a-*tce1-tci1* or pET28a-*tce1*$^{S8A/A16E}$ was shown as indicated. All micrographs were repeated for at least three times independently with similar results. DAPI, fluorescence observation; BF, bright field. Scale bars: 28 μm.

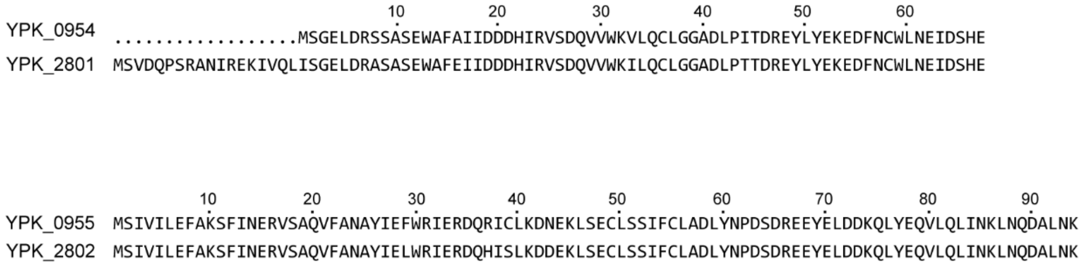

Supplementary Figure 3: Sequence alignment of YPK_0954 with YPK_2801 and YPK_0955 with YPK_2802. The protein sequence alignment was performed using ClustalX 2.1.

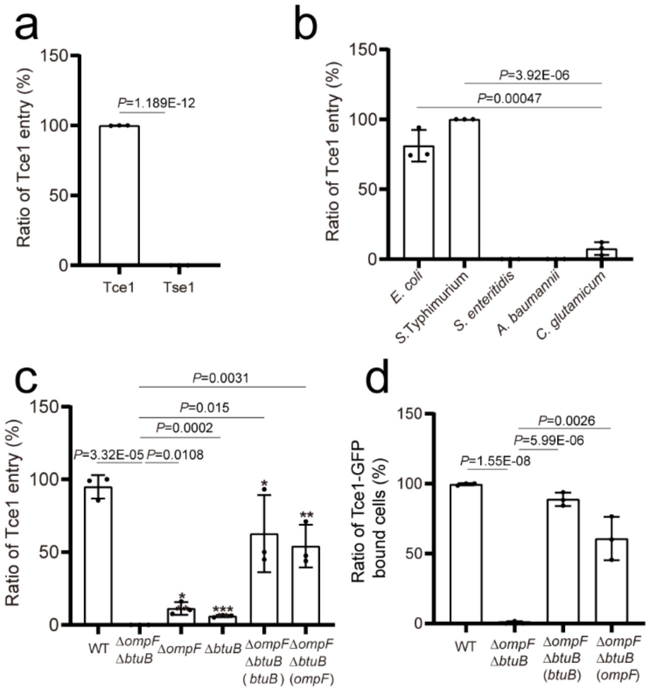

Supplementary Figure 4: Quantification of the percent of cells showing fluorescence above a relevant background intensity threshold. The threshold of fluorescence intensity was defined as 1.3 meaning Tce1 protein labelled cells only counted when fluorescence was 1.3-fold higher than background intensity. **a-c,** Based on this rule, the ratio of the Tce1 entry was calculated in **a**, **b** and **c** corresponding to **Fig.2c, Fig.2e** and **Fig.4a**, respectively. **d,** The ratio of Tce1-GFP bound cells was calculated corresponding to **Fig.4b**. All data were obtained from three independent experiments. Data are mean ± SD. Two-sided, unpaired Student's *t*-test was used for these analyses, and *P* values < 0.05 were considered to indicate statistically significant differences.

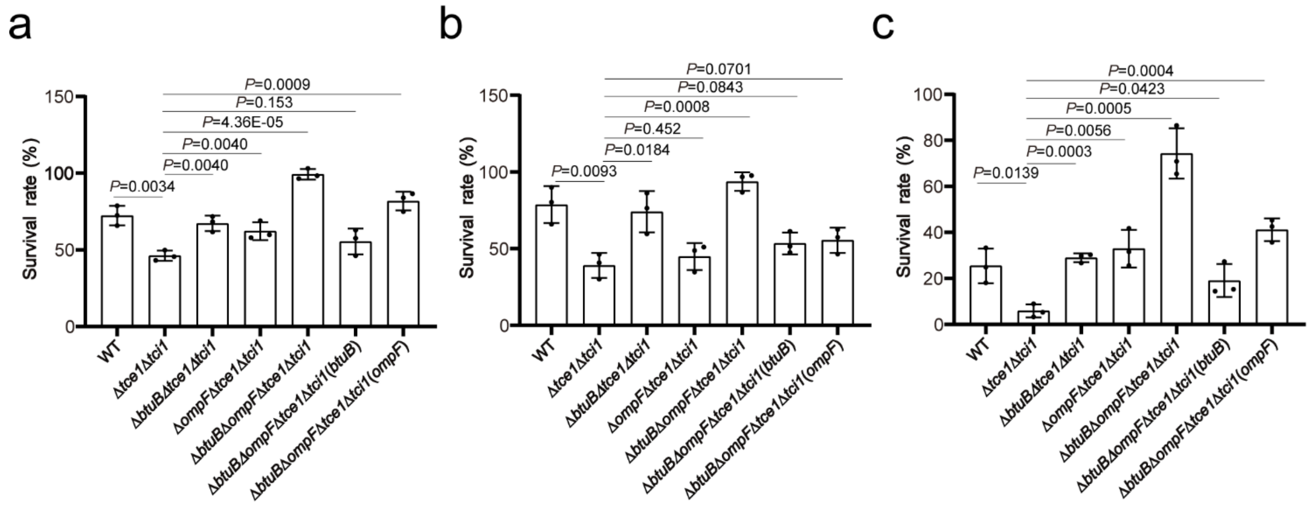

Supplementary Figure 5: Toxicity assays of purified Tce1 protein on killing of *Yptb* strains. The indicated *Yptb* strains were diluted 40-fold in M9 medium and treated with 0.005 **(a)**, 0.01 **(b)** or 0.1 **(c)** mg ml^{-1} of purified Tce1, respectively, for 1 h, and the viability of cells was determined by counting the CFU after treatment. All data were obtained with three independent experiments. Data are mean ± SD. Two-sided, unpaired Student's *t*-test was used for these analyses, and *P* values < 0.05 were considered to indicate statistically significant differences.

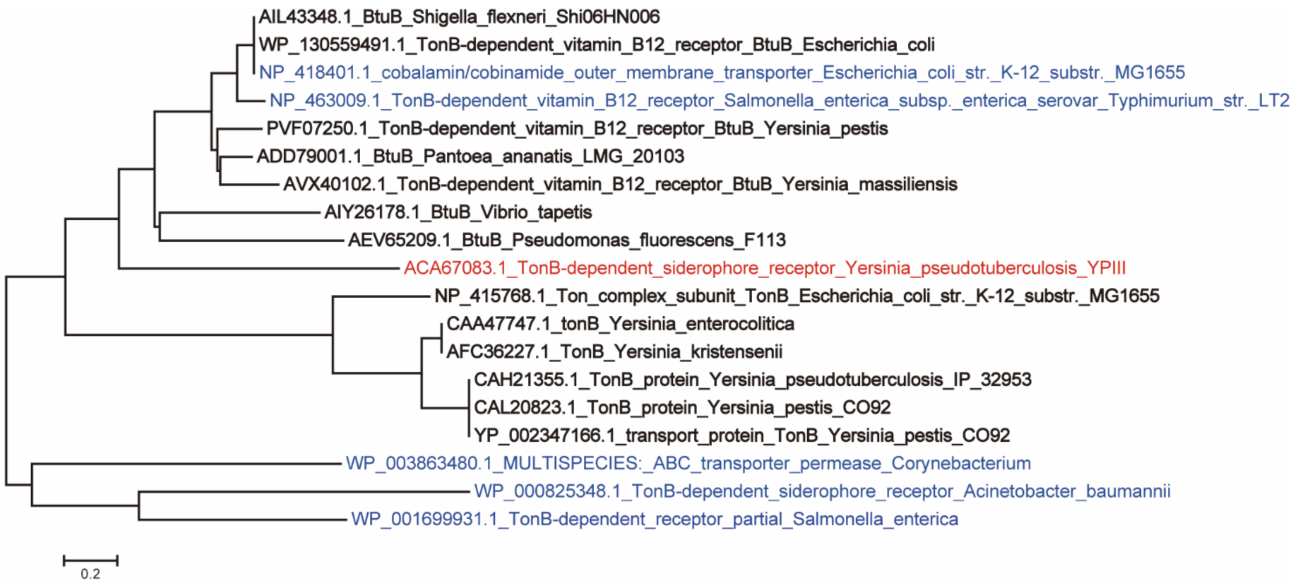

Supplementary Figure 6: Phylogenetic relationship of BtuB from different species. Different protein sequences were obtained from the Uniprot database (http://www.uniprot.org/). The phylogenetic tree was constructed using MEGA 7.0 by the neighbor-joining method and multiple sequence alignment was performed using ClustalX 2.1. The scale bar indicates percentage of divergence (distance). The *Y. pseudotuberculosis* YPIII YPK_0782 was indicated in red. The ortholog genes in *E. coli*, *S.* Typhimurium, *S. enteritidis*, *C. glutamicum*, and *A. baumannii* were indicated in blue.

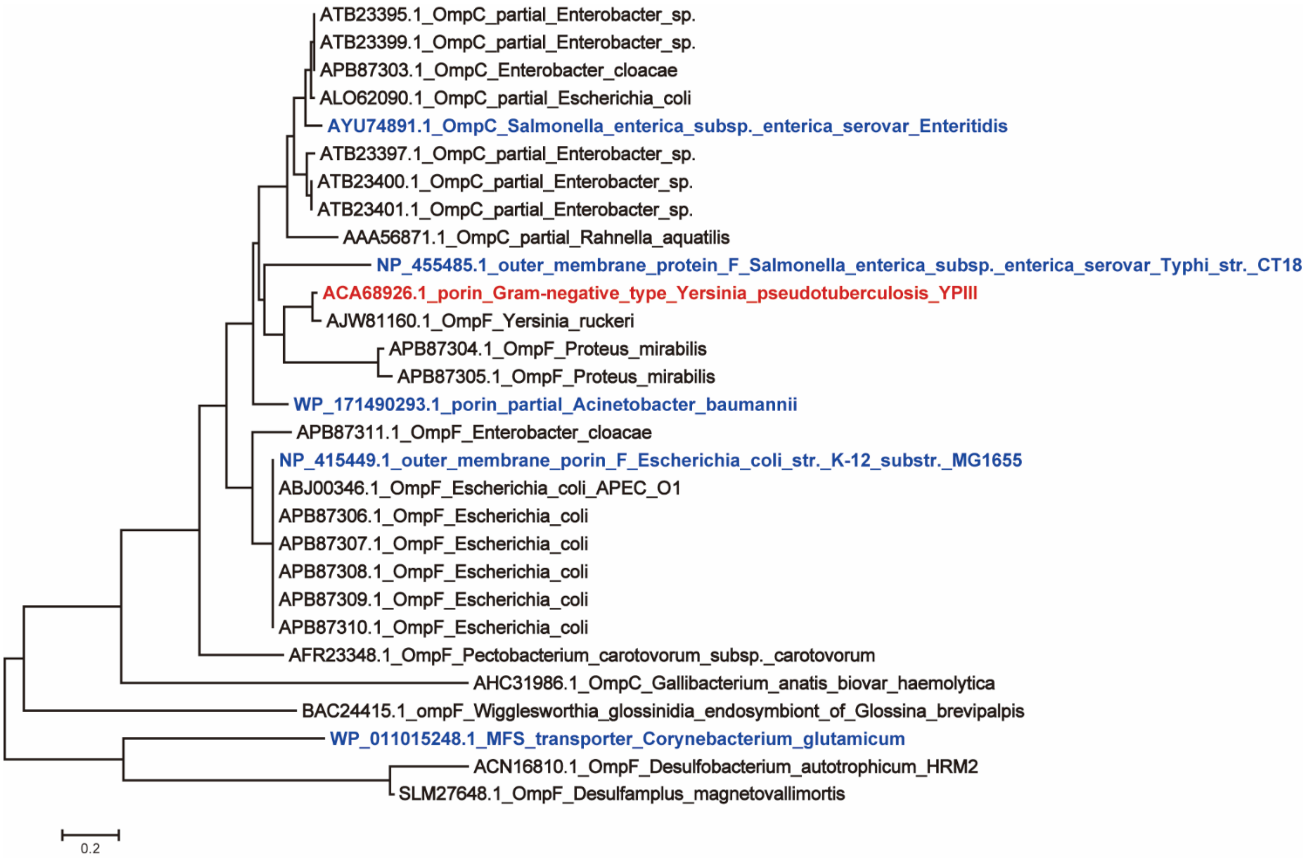

Supplementary Figure 7: Phylogenetic relationship of OmpF from different species. Different protein sequences were obtained from the Uniprot database (http://www.uniprot.org/). The phylogenetic tree was constructed using MEGA 7.0 by the neighbor-joining method and multiple sequence alignment was performed using ClustalX 2.1. The scale bar indicates percentage of divergence (distance). The *Y. pseudotuberculosis* YPIII YPK_2649 was indicated in red. The ortholog genes in *E. coli*, *S.* Typhimurium, *S. enteritidis*, *C. glutamicum*, and *A. baumannii* were indicated in blue.

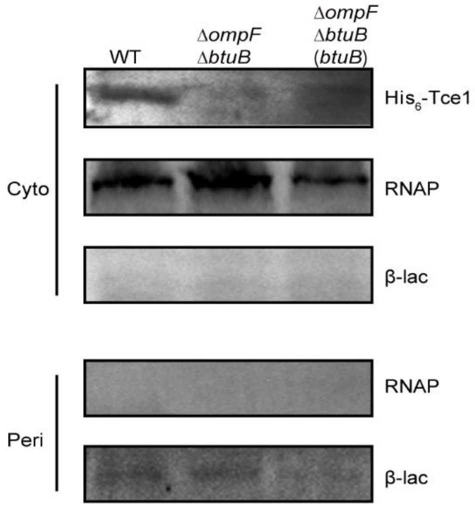

Supplementary Figure 8: Tce1 protein can enter into the cytosol of recipient cells. Purified Tce1 protein was added in the same condition of toxin assay. Then, periplasmic and cytosol proteins were isolated and examined by western blotting assay. RNAP was probed as control of the cytosol; β-lac (β-lactamase) was regarded as control of the periplasm. The result was repeated for three times independently with similar results.

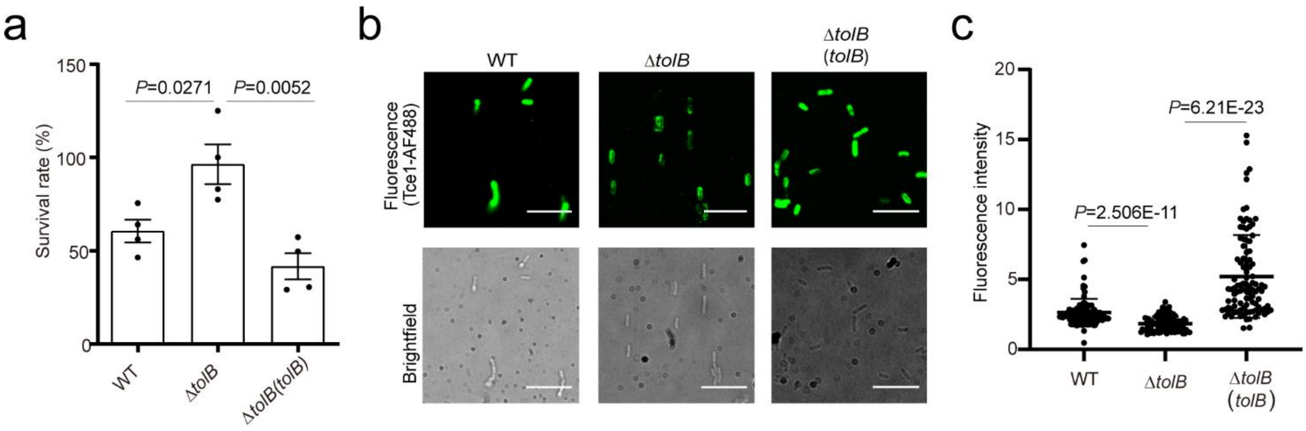

Supplementary Figure 9: Tce1 requires TolB for target cell entry. a, Toxicity assay of purified Tce1 protein to indicated strains. Indicated bacterial strains diluted 40-fold in M9 medium were treated by addition of purified Tce1 (0.1 mg ml^{-1}) for 1 h and the viability of cells was determined (n=4 independent experiments). **b,** Fluorescence labelling of indicated bacterial strains with Tce1-AF488. Scale bars, 20 μm. **c,** Quantification of the fluorescence intensity differences between indicated strains in **b**. Fluorescence intensity of 100 bacteria were counted based on the cell to background ratio. Both **a** and **c** are mean ± SD with two-sided, unpaired Student's *t*-test. Differences were considered statistically significant at *P* values < 0.05.

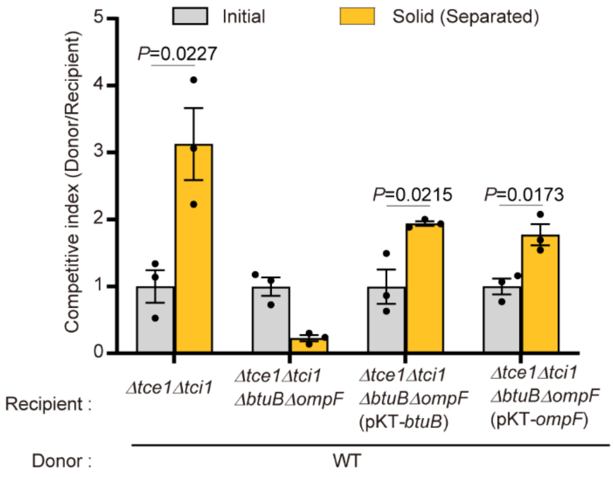

Supplementary Figure 10: Tce1 requires BtuB and OmpF in contact-independent competition. Contact-independent competition assay was performed by separating indicated donor and recipient cells with a cell-impermeable membrane and cultivated at 26°C for 48 h on the surface of M9 solid medium. The CFU ratio of the donor and recipient strains was counted by plate counts. Bars represent the mean donor : recipient CFU ratio from three independent experiments (± SD). Results were analyzed using two-sided, unpaired Student's *t*-test. Differences were considered statistically significant at *P* values < 0.05.

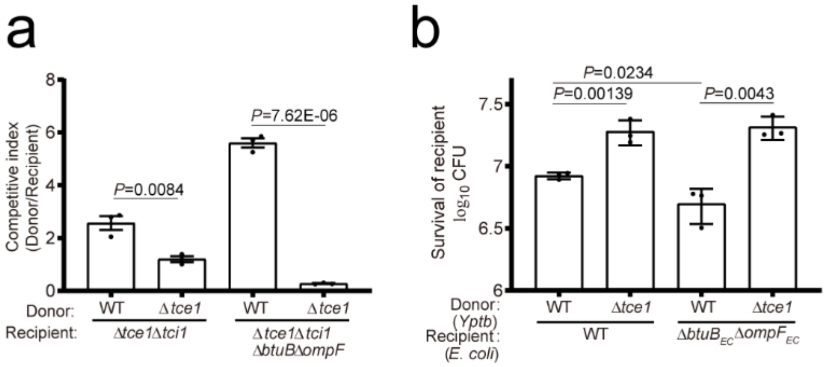

Supplementary Figure 11: BtuB and OmpF are not required for Tce1-mediated contact-dependent killing. a, Contact-dependent intra-species growth competition experiments between the indicated *Yptb* donor and Δ*tce1*Δ*tci1* or Δ*tce1*Δ*tci1*Δ*butB*Δ*ompF* recipient strains. Donor and recipient strains were mixed 1:1, and then grown for 48 h on a solid support at 26°C. The CFU ratio of the donor and recipient strains was measured by plate counts. Bars represent the mean donor : recipient CFU ratio from three independent experiments (± SD). **b,** Contact-dependent inter-species growth competition experiments between the indicated *Yptb* donor and *E. coli* DH5α or Δ*butB*$_{Ec}$Δ*ompF*$_{Ec}$ recipient strains. Donor and recipient strains were mixed 10:1, grown for 12 h on M9 plates at 26°C. The survival of *E*. *coli* cells was quantified by counting CFUs on selective plates. Bars represent the survival of *E. coli* from three independent experiments (± SD). Two-sided, unpaired Student's *t*-test. Differences were considered statistically significant at *P* values < 0.05.

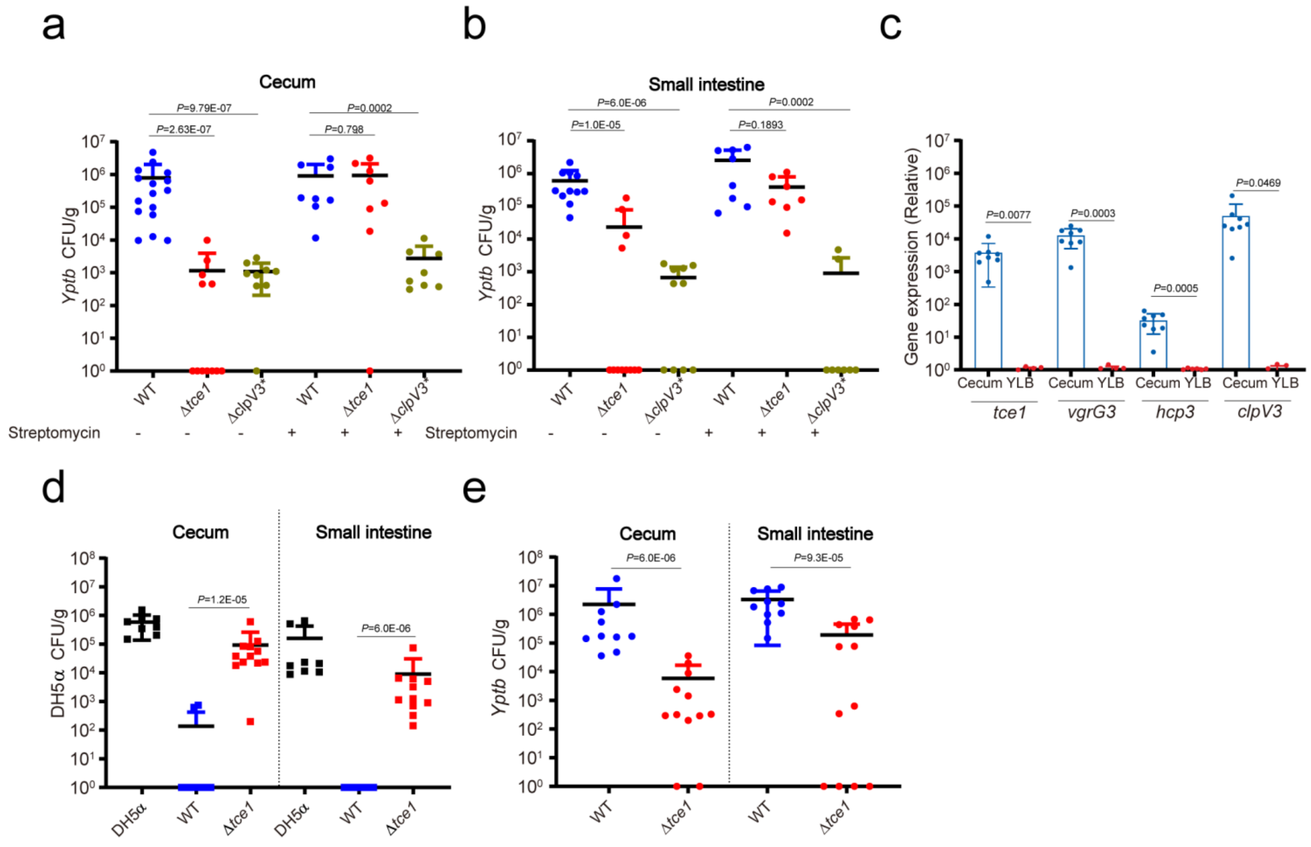

Supplementary Figure 12: Tce1-mediated T6SS killing pathway facilitates *Yptb* colonization of mouse gut. a-b, Mice pre-treated with or without streptomycin in **a** (Strep-: WT n=16; Δ*tce1* n=12; Δ*clpV3** n=9 and Strep+: WT n=8; Δ*tce1* n=8; Δ*clpV3** n=8) and **b** (Strep-: WT n=11; Δ*tce1* n=12; Δ*clpV3** n=10 and Strep+: WT n=8; Δ*tce1* n=7; Δ*clpV3** n=8) were orally gavaged with 10^9 CFUs of indicated *Yptb* strains. Animals were sacrificed 48 h after challenge, and bacterial loads in the cecum (**a**) and small intestine (**b**) were measured. **c,** The expression of *tce1* and T6SS-3 genes was induced during infection. 6-week-old female BALB/c mice (n=8) were orally gavaged with 10^9 CFUs of *Yptb* YPIII. Total RNA was extracted from cecum 24 h post infection and qRT-PCR was used to examine the expression of *tce1* and relevant T6SS-3 genes (*clpV3*, *vgrG3*, *hcp3*). Bacteria grown in YLB were used as controls. Data shown were the average of three independent experiments; error bars indicate SD from three independent experiments. Statistics analyses were performed by Student's *t*-test. **d-e,** Streptomycin-treated Mice (buffer control: n=8; WT: n=10; Δ*tce1*: n=12) were colonized with 5×10^8 CFUs of *E. coli* for 24 h and then challenge with 5×10^8 CFUs of *Yptb* WT, Δ*tce1* or buffer control. Animals were sacrificed 48 h after challenge and surviving *E. coli* (**d**) and *Yptb* (**e**) from cecum and small intestine were counted. PBS mixed with *E. coli* was regarded as negative control. Each data point represents result from one mouse; error bars represent mean ± SD of recovered CFUs. Statistical analysis of all experiments was carried out using the two-sided Mann-Whitney test. Differences were determined as significant when *P* value <0.05.

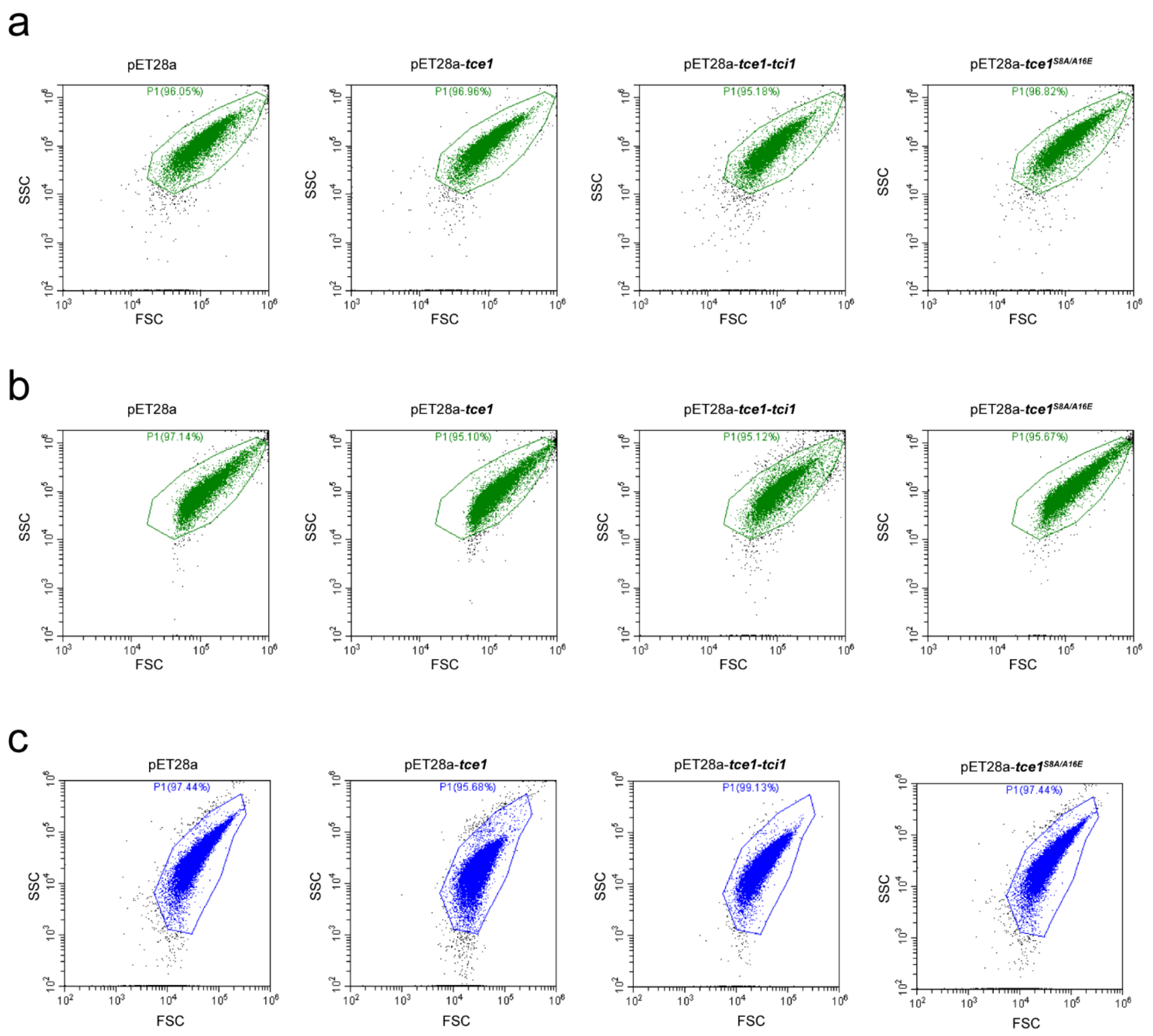

Supplementary Figure 13: **Figures for graphically account for FACS sequential gating of Figure 1 i and j. a-b,** the graphs are the gating for FITC in Figure 1i (**a**, 0 h; **b**, induced by IPTG for 4 h). **c**, the graph is for DAPI in Figure 1j. The bacterial morphs and sizes in each graph of same assay were similar and gated in same strategy. P1 in graphs represent the percentage of bacterial cells that passed the gating criteria.

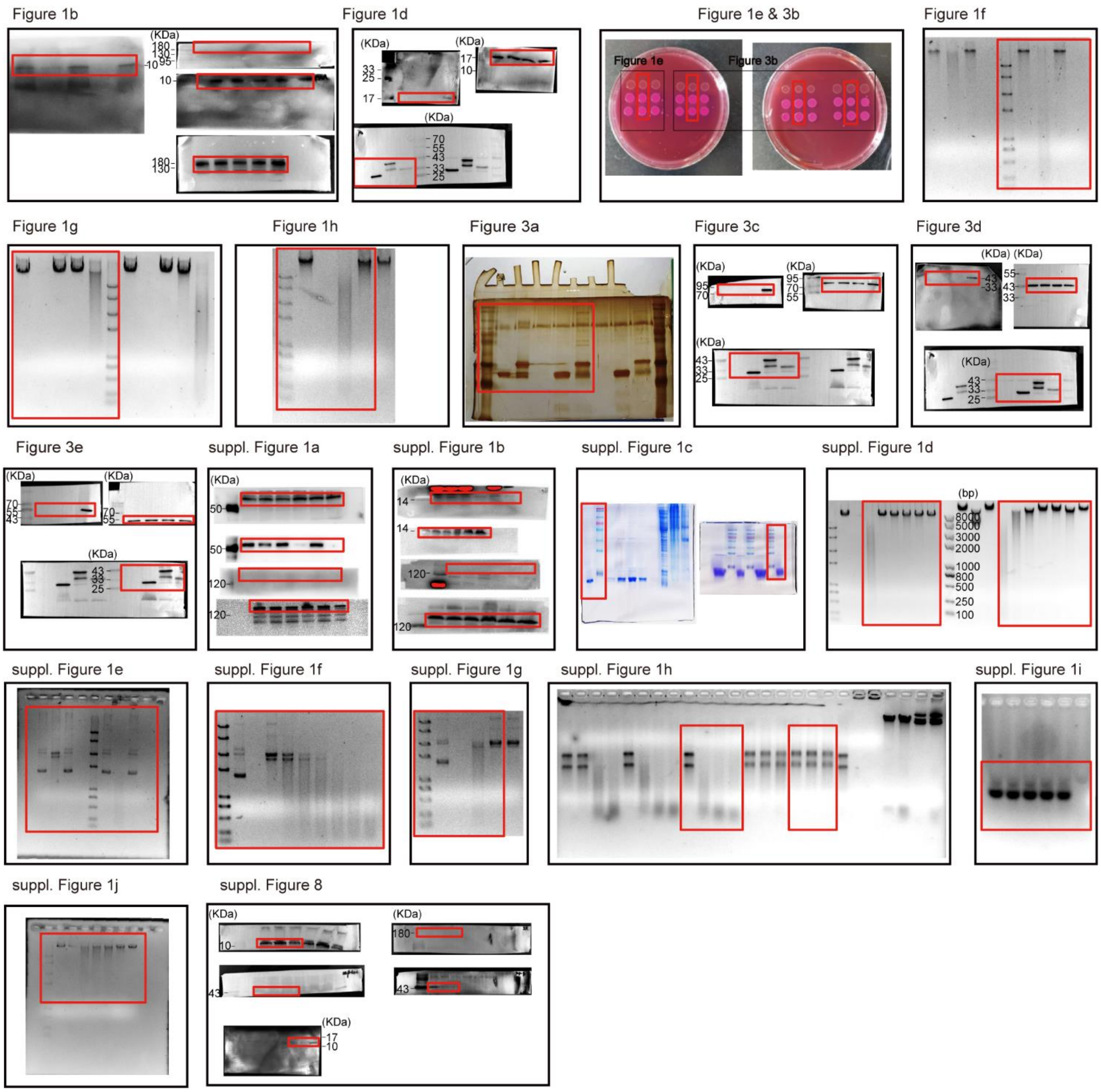

Supplementary Figure 14：Uncropped blots and gels.

Supplementary Table 1. Bacterial strains and plasmids used in this study.

Strains or plasmids	Relevant characteristics	Reference
E. coli		
S17-1*λ pir*	*λ*-pir lysogen of S17-1, *thi pro hsdR hsdM*$^+$ *recA* RP4 2-Tc::Mu-Km::Tn7	1
BL21(DE3)	Host for expression vector pET28a and toxin assay	Novagen
XL1 Blue	Host for expression vector pGEX6p-1	Novagen
BTH101	Host for bacterial two-hybrid	Novagen
DH5α	For competition assay	Beyotime
Δ*btuB*$_{Ec}$	*butB*$_{Ec}$ deleted in *E. coli* DH5α	This study
Δ*ompF*$_{Ec}$	*ompF*$_{Ec}$ deleted in *E. coli* DH5α	This study
Δ*btuB*$_{Ec}$Δ*ompF*$_{Ec}$	*butB*$_{Ec}$ and *ompF*$_{Ec}$ deleted in *E. coli* DH5α	This study
Yersinia pseudotuberculosis		
YPIII	Wild-type *Y. pseudotuberculosis* pIB1, Nal^R	2
Δ*clpV1*	*clpV1* deleted in *Y. pseudotuberculosis* YPIII	2
Δ*clpV2*	*clpV2* deleted in *Y. pseudotuberculosis* YPIII	2
Δ*clpV3*	*clpV3* deleted in *Y. pseudotuberculosis* YPIII	2
Δ*clpV4*	*clpV4* deleted in *Y. pseudotuberculosis* YPIII	2
Δ4*clpV*	*clpV1, clpV2, clpV3* and *clpV4* deleted in *Y. pseudotuberculosis* YPIII	2
**Δ*ypk_2801*Δ*ypk_2802* (WT)**	*ypk_2801* and *ypk_2802* deleted in *Y. pseudotuberculosis* YPIII, considered as the wild-type background in this study	This study
Δ*tce1*Δ*tci1*	*tec1* and *tci1* deleted in Δ*ypk_2801*Δ*ypk_2802*	This study
Δ*tce1*	*tec1* deleted in Δ*ypk_2801*Δ*ypk_2802*	This study
Δ*clpV3**	*clpV3* deleted in Δ*ypk_2801*Δ*ypk_2802*	This study
Δ*btuB*	*btuB* deleted in Δ*ypk_2801*Δ*ypk_2802*	This study
Δ*ompF*	*ompF* deleted in Δ*ypk_2801*Δ*ypk_2802*	This study
Δ*btuB*Δ*ompF*	*btuB* and *ompF* deleted in Δ*ypk_2801*Δ*ypk_2802*	This study
Δ*btuB*Δ*tce1*Δ*tci1*	*btuB*, *tec1* and *tci1* deleted in Δ*ypk_2801*Δ*ypk_2802*	This study
Δ*ompF*Δ*tce1*Δ*tci1*	*ompF*, *tec1* and *tci1* deleted in Δ*ypk_2801*Δ*ypk_2802*	This study
Δ*btuB*Δ*ompF*Δ*tce1*Δ*tci1*	*btuB*, *ompF*, *tec1* and *tci1* deleted in Δ*ypk_2801*Δ*ypk_2802*	This study
**Plasmid**		
pME6032	Shuttle vector, Tc^R	3
pME6032-*tce1-vsvg*	*tce1-vsvg* under the control of chloramphenicol resistance gene promoter in plasmid pME6032	This study
pME6032-*ypk_0952-vsvg*	*ypk_0952-vsvg* under the control of chloramphenicol resistance gene promoter in plasmid pME6032	This study
pET28a	Expression vector with N-terminal hexahistidine affinity tag, Km^R	Novagen
pET28a-*tce1*	pET28a carrying *tce1* coding region, Km^R	This study
pET28a-*tce1-gfp*	pET28a carrying *tce1* and *gfp* coding region, Km^R	This study
pET28a-*tce1-tci1*	pET28a carrying *tce1* and *tci1* coding region, Km^R	This study
pET28a-*tce1*$^{S8A/A16E}$	pET28a carrying *tce1*^{S8AA16E} coding region, Km^R	This study
pGEX6p-1	Expression vector with N-terminal GST tag, Amp^R	Novagen
pGEX6p-1-*tce1*	pGEX6p-1 carrying *tce1* coding region, Amp^R	This study
pDM4	Suicide vector, *mob*RK2, *ori*R6K, *pir*, *sacB*, Cm^R	4
pDM4-Δ*ypk_2801-2802*	Construct used for in-frame deletion of *ypk_2801*and *ypk_2802*, Cm^R	This study
pDM4-Δ*tce1*	Construct used for in-frame deletion of *tce1,* Cm^R	This study

pDM4-Δ*tce1*Δ*tci1*	Construct used for in-frame deletion of *tce1, tci1*, Cm^{R}	This study
pDM4-Δ*btuB*	Construct used for in-frame deletion of *btuB*, Cm^{R}	This study
pDM4-Δ*ompF*	Construct used for in-frame deletion of *ompF*, Cm^{R}	This study
pKT100	Cloning vector, p15A replicon, Km^{R}	5
pKT100-*tce1*	*tce1* under the control of chloramphenicol resistance gene promoter in plasmid pKT100	This study
pKT100-*tci1*	*tci1* under the control of chloramphenicol resistance gene promoter in plasmid pKT100	This study
pKT100-*btuB*	*btuB* under the control of chloramphenicol resistance gene promoter in plasmid pKT100	This study
pKT100-*ompF*	*ompF* under the control of chloramphenicol resistance gene promoter in plasmid pKT100	This study
pKT100-*mcherry*	*mcherry* under the control of chloramphenicol resistance gene promoter in plasmid pKT100	This study
pKT25	p15A origin of replication encoding CyaA$_{1-224}$; Km^{R}	6
pUT18C	ColE1 origin of replication encoding CyaA$_{225-399}$; Amp^{R}	6
pKT25-*tce1*	*tce1* in pKT25	This study
pUT18C-*tci1*	*tci1* in pUT18C	This study
pUT18C-*btuB*	*btuB* in pUT18C	This study
pUT18C-*ompF*	*ompF* in pUT18C	This study
pKT25-*zip*	Leucine zipper of GCN1 (BTH positive control); Km^{R}	6
pUT18C-*zip*	Leucine zipper of GCN1 (BTH positive control); Amp^{R}	6
pCas	CRISPR-Cas9 system plasmid used for in-frame deletion, Km^{R}	7
pTargetF1	pTargetF with spectinomycin resistance gene replaced by a chloramphenicol resistance gene, Cm^{R}	8
pTargetF1-Δ*btuB$_{Ec}$*	Construct used for in-frame deletion of *btuB* in *E. coli*	This study
pTargetF1-Δ*ompF$_{Ec}$*	Construct used for in-frame deletion of *ompF* in *E. coli*	This study
pBBRMCS5	Cloning vector containing REP, Gm^{R}	9
pBBRMCS5-*gfp*	*gfp* in pBBRMCS5	This study
pACYC184	Cloning vector, p15A origin of replication, Cm^{R}	10

* Nal^{R}, Cm^{R}, Km^{R}, Tc^{R}, Gm^{R}, and Amp^{R} represent resistance to naladixic acid, chloramphenicol, kanamycin, tetracycline and ampicillin, respectively.

Supplementary Table 2. Primers used in this study.

Primers	5'-3' sequence	Function
tce1-M1F-*Bam*HI	GTGCGGATCCAGAAAATAACTCCGGTTCAAAATG	To generate
tce1-M1R	GCGGTCAAGTTCCCCTGAC	pDM4-Δ*tce1*
tce1-M2F	**GTCAGGGGAACTTGACCGC**GAAATTGACTCACATGAGTAATT	
tce1-M2R-*Sal*I	CTGCGTCGACACAGCCAGTATTATCAGAAGAAGC	
tci1-M2F	**GTCAGGGGAACTTGACCGC**CAGGACGCTCTAAATAAATAGCC	To generate pDM4-Δ*tec1*Δ*tci1*
tci1-M2R-*Sal*I	CTGCGTCGACTATGCCGTGCTTCTCTATATCG	
ypk_2801-M1F-*Bgl*II	GGAAGATCTCCTTATCAATATGCGGGAACGTG	To generate pDM4-
ypk_2801-M1R	CTGGTCTACTGACATTTTATCTTCTCC	Δ*ypk_2801-2802*
ypk_2802-M2F	**GGAGAAGATAAAATGTCAGTA**GACGCTCTAAATAAATAGCATATTGAC	
ypk_2802- M2R-*Sal*I	ACGCGTCGACAGAGTATAGATGTTATGAGGGGAGG	
ompF-M1F-*Sph*I	ACATGCATGCTGCAACGTTTCGTCACCTCTGAT	To generate
ompF-M1RF	AAGAATATTGCGCTTCATCATTATTAT	pDM4-Δ*ompF*
ompF-M2F	**ATAATAATGATGAAGCGCAATATTCTT**GGCTTGGTTTACCAGTTCTAAGTCAAAC	
ompF-M2R-*Sal*I	ACGCGTCGACGGCAGCAGCCGTGGAACAATACC	
btuB-M1F-*Bgl*II	GGAAGATCTTTTCTCGCTGCATGGGGG	To generate
btuB-M1R	GTGTTTGTGTTTCATTGTGACTTTCT	pDM4-Δ*btuB*
btuB-M2F	AGAAAGTCACAATGAAACACAAACACCAGTGTTGTTCTGATAGATAGTGTTGTTC	
btuB-M2R-*Sal*I	ACGCGTCGACTTTAGCTTGTCTGGGAACGCCAT	
b3966-g20-F1-*Spe*I	AGCTAGCTCAGTCCTAGGTATAATACTAGTTTTTTAAGTAAAACGCTTTAGTTTTAGAGCTAGAAATAGC	pTargetF1-Δ*btuB*$_{Ec}$
b3966-g20-R	CTCAAAAAAAGCACCGACTCGG	
b3966-up-F	CCGAGTCGGTGCTTTTTTTGAGGCGATTGTAGGGATTGCTC	
b3966-up-R	CAGTAACGACGAGAGTATCCG	
b3966-down-F	CGGATACTCTCGTCGTTACTGTGACAGTTCGTGGTAAAATAGC	
b3966-down-R-*Sal*I	GGTAATAGATCTAAGCTTCTGCAGGTCGACGCCATTTGCCGTCAGAC	
b0929-g20-F1-*Spe*I	AGCTAGCTCAGTCCTAGGTATAATACTAGTTACAGCGA	pTargetF1-Δ*ompF*$_{Ec}$
b0929-g20-R	CTCAAAAAAAGCACCGACTCGG	
b0929-up-F	CCGAGTCGGTGCTTTTTTTGAGTCTTTGTAGCACTTTCACGGTA	
b0929-up-R	CACCGTAACTGTTTTCACCG	
b0929-down-F	CGGTGAAAACAGTTACGGTGCTTACACCAAATCTAAAGCGAA	
b0929-down-R-*Sal*I	GGTAATAGATCTAAGCTTCTGCAGGTCGACGAAGTCCGCTATCAGGGTAA	
tce1-F-*Bam*HI	GTGCGGATCCATGTCAGGGGAACTTGACCG	To generate
tce1-R-*Sal*I	GTGCGTCGACTTACTCATGTGAGTCAATTTCGT	pET28a-*tce1*
tci1-F-*Eco*RI	CCGGAATTCATGAGCATAGTTATTTTAGAATTCGC	To generate
tci1-R-*Bgl*II	GTGAAGATCTCTATTTATTTAGAGCGTCCTGATTTA	pME6032-*tci1*
tce1-F-*Bam*HI	CGCGGATCCATGTCAGGGGAACTTGACCG	To generate
tce1-R-*Eco*RI(TAA)	CGGAATTCTCATGTGAGTCAATTTCGT	pET28a-*tce1*-*GFP*
GFP-F-*Eco*RI	CGGAATTCATGGTGAGCAAGGGCGAGG	and pBBRMCS5-*GFP*
GFP-F-*Sal*I	ACGCGTCGACTTACTTGTACAGCTCGTCCATGCCG	
tce1-F-*Bam*HI	CGCGGATCCATGTCAGGGGAACTTGACCG	To generate
tce1-R-*Sal*I	CTGCGTCGACTTACTCATGTGAGTCAATTTCGTTG	pKT100-*tce1*
tci1-F-*Bam*HI	GTGCGGATCCATGAGCATAGTTATTTTAGAATTCG	To generate

tci1-R-*Sal*I	GTGCGTCGACCTATTTATTTAGAGCGTCCTGATT	pKT100-*tci1*
btuB-F-*Bam*HI	GTGCGGATCCATGAAACACAAACACCTCTGG	To generate
btuB-R-*Sal*I	GTGCGTCGACTCAGAACAACACTGAGTAGTTCA	pKT100-*btuB*
ompF-F-*Bam*HI	GTGCGGATCCATGATGAAGCGCAATATTCTTGC	To generate
ompF-R-*Sal*I	GTGCGTCGACTTAGAACTGGTAAACCAAGCCAAC	pKT100-*ompF*
b3966-F-*Bam*HI	CGCGGATCCATGATTAAAAAAGCTTCGCTGCTG	To generate
b3966-R-*Xho*I	CCGCTCGAGTCAGAAGGTGTAGCTGCCAGACA	pKT100-*btuB*$_{Ec}$
b0929-F-*Bam*HI	CGCGGATCCATGATGAAGCGCAATATTCTGGC	To generate
b0929-R-*Sal*I	ACGCGTCGACTTAGAACTGGTAAACGATACCCACAGC	pKT100-*ompF*$_{Ec}$
tce1-F-*Bam*HI	CTGAGGATCCATGTCAGGGGAACTTGACCG	To generate
tce1-R-*Sal*I	GTGCGTCGACTTACTCATGTGAGTCAATTTCGT	pGEX6p-1-*tce1*
tce1-F-*Eco*RI	CCGGAATTCATGTCAGGGGAACTTGACC	To generate
tce1-R-VSVG-*Bgl*II	GTGAAGATCTTCATTTTCCTAATCTATTCATTTCAATATCTGTA TACTCATGTGAGTCAATTTCGT	pME6032-*tce1-vsvg*
0952-FM-F-*Eco*RI	CCGGAATTCATGGCAAAAGGTTATTATCTGGT	To generate
0952-FM-R-*Bgl*II	GAAGATCTTCATTTTCCTAATCTATTCATTTCAATATCTGTATA TTGATCGTTCCAATAGTCC	pME6032-*ypk0952-vsvg*
btuB-pUT18C-F-*Xba*I	GCTCTAGAGATGAAACACAAACACCTCTGGGT	To generate
btuB-pUT18C -R-*Kpn*I	CGGGGTACCTCAGAACAACACTGAGTAGTTCACACC	pUT18C-*btuB*
ompF-pUT18C-F-*Xba*I	GCTCTAGAGATGATGAAGCGCAATATTCTTGC	To generate
ompF-pUT18C-R-*Kpn*I	CGGGGTACCTTAGAACTGGTAAACCAAGCCAA	pUT18C-*ompF*
tci1-pUT18C-F-*Xba*I	GCTCTAGAGATGAGCATAGTTATTTTAGAATTCG	To generate
tci1-pUT18C -R-*Kpn*I	CGGGGTACCCTATTTATTTAGAGCGTCCTGATT	pUT18C-*tci1*
tce1-t25-F-*Bam*HI	CGCGGATCCCATGTCAGGGGAACTTGACC	To generate
tce1-t25-R-*Eco*RI	CCGGAATTCTTACTCATGTGAGTCAATTTCGT	pKT25-*tce1*
16S RNA-F	CTAGCGATTCCGACTTCAT	qRT-PCR
16S RNA-R	CCCTTATCCTTTGTTGCC	
clpV3-F	GCTTCATTCGCTTCTGGT	
clpV3-R	GTGGCTATTACAAGGAAACATCT	
hcp3-F	ATGTGCCTCCGTATTTATATGGG	
hcp3-R	GTCACTCAATGCCAAACA	
vgrG3-F	GTTGGAAGGCGAGGAAGC	
vgrG3-R	GGCTCACCCAATAGATAAGGCAT	
tce1-F	CCTCTGAATGGGCGTTTG	
tce1-R	ATCGGTAAATCTGCTCCC	

Underlined sites indicate restriction enzyme cutting sites added for cloning. Letters in boldface denote the annealing regions for overlap PCR.

Supplementary References

1. Simon R. *et al.* A broad host range mobilization system for in vivo genetic engineering: transposon mutagenesis in gram negative bacteria. *Nat Biotechnol* **1**, 784–791 (1983)
2. Wang, T. *et al.* Type VI secretion system transports Zn^{2+} to combat multiple stresses and host immunity. *PLoS Pathog* **11**, e1005020 (2015)
3. Heeb, S., Blumer, C. & Haas, D. Regulatory RNA as mediator in GacA/RsmA-dependent global control of exoproduct formation in *Pseudomonas fluorescens* CHA0. *J Bacteriol* **184**, 1046-1056 (2002)
4. Zhao Y. *et al.* The NAIP-NLRC4 inflammasome in innate immune detection of bacterial flagellin and type III secretion apparatus. *Immunol Rev* **265(1)**, 85-102 (2015)
5. Hu, Y. *et al.* OmpR positively regulates urease expression to enhance acid survival of *Yersinia pseudotuberculosis*. *Microbiology* **155**, 2522-2531 (2009).
6. Karimova, G., Pidoux, J., Ullmann, A. & Ladant, D. A bacterial two-hybrid system based on a reconstituted signal transduction pathway. *Proc Natl Acad Sci U S A* **95**, 5752-5756 (1998).
7. Jiang, Y. *et al.* Multigene editing in the Escherichia coli genome via the CRISPR-Cas9 system. *Appl Environ Microbiol* **81**, 2506-2514 (2015)
8. Zhang, L. *et al.* The Catabolite repressor/activator Cra is a bridge connecting carbon metabolism and host colonization in the plant drought resistance-promoting bacterium *Pantoea alhagi* LTYR-11Z. *Appl Environ Microbiol* **84** (2018).
9. Kovach, M. E. *et al.* Four new derivatives of the broad-host-range cloning vector pBBR1MCS, carrying different antibiotic-resistance cassettes. *Gene* **166**, 175-176 (1995).
10. Chang, A. C. & Cohen, S. N. Construction and characterization of amplifiable multicopy DNA cloning vehicles derived from the P15A cryptic miniplasmid. *J Bacteriol* **134**, 1141-1156 (1978).
