# Supplementary material for: Contact-independent killing mediated by a T6SS effector with intrinsic cell-entry properties
Source: Nat Commun. 2021 Jan 18;12:423. doi: 10.1038/s41467-020-20726-8 (PMC7813860; doi:10.1038/s41467-020-20726-8)
Supplement: Supplementary file 3 — Reporting Summary [file 41467_2020_20726_MOESM3_ESM.pdf]

nature research

Corresponding author(s): Xihui Shen & Yao Wang

Last updated by author(s): Dec 7, 2020

Reporting Summary

Nature Research wishes to improve the reproducibility of the work that we publish. This form provides structure for consistency and transparency in reporting. For further information on Nature Research policies, see our Editorial Policies and the Editorial Policy Checklist.

Statistics

For all statistical analyses, confirm that the following items are present in the figure legend, table legend, main text, or Methods section.

n/a	Confirmed	
☐	☒	The exact sample size (*n*) for each experimental group/condition, given as a discrete number and unit of measurement
☐	☒	A statement on whether measurements were taken from distinct samples or whether the same sample was measured repeatedly
☐	☒	The statistical test(s) used AND whether they are one- or two-sided<br>*Only common tests should be described solely by name; describe more complex techniques in the Methods section.*
☒	☐	A description of all covariates tested
☒	☐	A description of any assumptions or corrections, such as tests of normality and adjustment for multiple comparisons
☐	☒	A full description of the statistical parameters including central tendency (e.g. means) or other basic estimates (e.g. regression coefficient) AND variation (e.g. standard deviation) or associated estimates of uncertainty (e.g. confidence intervals)
☐	☒	For null hypothesis testing, the test statistic (e.g. *F*, *t*, *r*) with confidence intervals, effect sizes, degrees of freedom and *P* value noted<br>*Give P values as exact values whenever suitable.*
☒	☐	For Bayesian analysis, information on the choice of priors and Markov chain Monte Carlo settings
☒	☐	For hierarchical and complex designs, identification of the appropriate level for tests and full reporting of outcomes
☒	☐	Estimates of effect sizes (e.g. Cohen's *d*, Pearson's *r*), indicating how they were calculated

Our web collection on statistics for biologists contains articles on many of the points above.

Software and code

Policy information about availability of computer code

Data collection	Beckman, CytoFLEX for flowcytometry, Andor Revolution-XD high-speed rotary disc type fluorescence confocal microscope, Tanon 5200Multi Chemiluminescence imager, Bio-Rad CFX96 Real-Time PCR Detection System, Thermo Fisher Scientific NanoDrop 2000, Uniport database, KEGG.
Data analysis	GraphPad Prism 7.0, NanoAnalyze_3.4, MEGA 7.0, CLUSTAL X v2.1, Gel Analysis 4.2, FlowJo_V10, For fluorescence microscopy analyses: ImageJ v1.48.

For manuscripts utilizing custom algorithms or software that are central to the research but not yet described in published literature, software must be made available to editors and reviewers. We strongly encourage code deposition in a community repository (e.g. GitHub). See the Nature Research guidelines for submitting code & software for further information.

Data

Policy information about availability of data

All manuscripts must include a data availability statement. This statement should provide the following information, where applicable:

- Accession codes, unique identifiers, or web links for publicly available datasets
- A list of figures that have associated raw data
- A description of any restrictions on data availability

The protein sequences are available from the Uniprot database (http://www.uniprot.org/). The source data underlying Fig. 1c, 1e, 2a–b, 2d, 2f-g, 3b, 4c–e, 5a-f and Supplementary Fig. 4a-d, 5a-c, 9a, 9c, 10, 11a-b, and 12a-e are provided as a Source Data file. Other data supporting the findings of this study are included in the article and its Supplementary Information files, or from the corresponding authors upon request.

Field-specific reporting

Please select the one below that is the best fit for your research. If you are not sure, read the appropriate sections before making your selection.

☒ Life sciences ☐ Behavioural & social sciences ☐ Ecological, evolutionary & environmental sciences

For a reference copy of the document with all sections, see nature.com/documents/nr-reporting-summary-flat.pdf

Life sciences study design

All studies must disclose on these points even when the disclosure is negative.

Sample size	We used 3-4 biological replicates for experiments as that is standard practice for most biochemical or microbiological assays.
Data exclusions	No data pertaining to any sample shown in this study was excluded.
Replication	All results reported in the paper were reliably reproduced in at least three independent experiments.
Randomization	All animals used were age, sex and vendor matched. Animals were randomly allocated to each group.
Blinding	Investigators were not blinded during data collection or analysis since there was not group allocation.

Reporting for specific materials, systems and methods

We require information from authors about some types of materials, experimental systems and methods used in many studies. Here, indicate whether each material, system or method listed is relevant to your study. If you are not sure if a list item applies to your research, read the appropriate section before selecting a response.

Materials & experimental systems

n/a	Involved in the study	
☐	☒	Antibodies
☒	☐	Eukaryotic cell lines
☒	☐	Palaeontology and archaeology
☐	☒	Animals and other organisms
☒	☐	Human research participants
☒	☐	Clinical data
☒	☐	Dual use research of concern

Methods

n/a	Involved in the study	
☒	☐	ChIP-seq
☐	☒	Flow cytometry
☒	☐	MRI-based neuroimaging

Antibodies

Antibodies used

mouse anti-VSV-G (F-6) (Supplier: Santa Cruz biotechnology, catalog no. sc-365019, lot number: B0916) validated by Western Blot in Yersinia pseudotuberculosis; mouse anti-ICDH (Xu L et al. Inhibition of host vacuolar H+-ATPase activity by a Legionella pneumophila effector. PLoS Pathog. (2010)) validated by Western Blot in Yersinia pseudotuberculosis; anti-RNAP (Supplier:(Santa Cruz biotechnology, catalog no. sc-56766, lot number: F2514) validated by Western Blot in Yersinia pseudotuberculosis; mouse anti-β-lactamase (Supplier: Santa Cruz biotechnology, catalog no. sc-66062, lot number: 8A5.A10) validated by Western Blot in Yersinia pseudotuberculosis; mouse anti-His (Supplier: Santa Cruz biotechnology, catalog number: sc-8036, lot number: I1018) validated by Western Blot in E.coli; mouse anti-GST (Supplier: Santa Cruz biotechnology, catalog number: sc-53909, lot number: F2413) validated by Western Blot in E.coli; horseradish peroxidase-conjugated Goat Anti-Mouse IgG (Supplier: Shanghai Genomics, catalog no. DY60203, lot number: 20614) validated by Western Blot in Yersinia pseudotuberculosis.

Validation

VSV-G (F-6) is a mouse monoclonal antibody specific for an epitope mapping between amino acids 493-511 at the C-terminus of VSV-G of Vesicular stomatitis virus origin.VSV-G (F-6) is recommended for detection of VSV-G of VSV origin by Western Blot (starting dilution 1:200, dilution range 1:100-1:1000), https://datasheets.scbt.com/sc-365019.pdf.

RNA pol β (8RB13) is a mouse monoclonal antibody raised against RNA pol β of E. coli origin. RNA pol β (8RB13) is recommended for detection of RNA pol β of E. coli origin by Western Blot (starting dilution 1:200), https://datasheets.scbt.com/sc-56766.pdf.

β lactamase (8A5.A10) is a mouse monoclonal antibody raised against 5'-His-tagged TEM1 β lactamase, lactamase (8A5.A10) is recommended for detection of TEM-type β lactamases of gram negative bacteria origin by Western Blot (starting dilution 1:200, dilution range 1:100-1:1000), https://datasheets.scbt.com/sc-66062.pdf.

His-probe (H-3) is a mouse monoclonal antibody raised against a His tagged recombinant protein, His-probe (H-3) is recommended for detection of fusion proteins encoded by polyhistidine expression vectors origin by Western Blot (starting dilution 1:200), https://datasheets.scbt.com/sc-8036.pdf.

GST (1E5) is a mouse monoclonal antibody raised against recombinant Glutathione S-transferase.GST (1E5) is recommended for detection of GST by Western Blotting (starting dilution 1:200, dilution range 1:100-1:1000), https://datasheets.scbt.com/sc-53909.pdf.

The antibodies were validated by the supplier (see website) and also in our laboratory using Western Blot assays in Yersinia

pseudotuberculosis and are cited in our labs previous publications.
-Xu L et al. Inhibition of host vacuolar H+-ATPase activity by a Legionella pneumophila effector. PLoS Pathog. (2010).
-Wang T et al. Type VI Secretion System Transports Zn2+ to Combat Multiple Stresses and Host Immunity. PLoS Pathog. (2015).
-Si M et al. Manganese scavenging and oxidative stress response mediated by type VI secretion system in Burkholderia thailandensis. Proc Natl Acad Sci U S A. (2017).

Animals and other organisms

Policy information about studies involving animals; ARRIVE guidelines recommended for reporting animal research

Laboratory animals	Six-week-old BALB/c female mice were maintained in mini-isolators housed in ventilated racks with controlled conditions (temperature of 24±2 °C, 50±10% humidity, air flow of 35 exchanges/hour and 12-h light/12-h dark cycle) with free access to food and water.
Wild animals	Study did not involve wild animals
Field-collected samples	Study did not involve field-collected samples.
Ethics oversight	All mouse experimental procedures were performed in accordance with the Regulations for the Administration of Affairs Concerning Experimental Animals approved by the State Council of People's Republic of China. The protocol was approved by the Animal Welfare and Research Ethics Committee of Northwest A&F University (protocol number: NWAFUSM2018001).

Note that full information on the approval of the study protocol must also be provided in the manuscript.

Flow Cytometry

Plots

Confirm that:

- [x] The axis labels state the marker and fluorochrome used (e.g. CD4-FITC).
- [x] The axis scales are clearly visible. Include numbers along axes only for bottom left plot of group (a 'group' is an analysis of identical markers).
- [x] All plots are contour plots with outliers or pseudocolor plots.
- [x] A numerical value for number of cells or percentage (with statistics) is provided.

Methodology

Sample preparation	Overnight culture of E. coli BL21(DE3) containing the pET28a plasmid or its derivatives expressing Tce1 alone (pET28a-tce1) or Tce1-Tci1 together (pET28a-tce1-tci1) were diluted 100-fold into LB broth and incubated at 26°C with 180 rpm shaking. After incubated at 26°C for 2 h, the expression of toxin and immunity genes was induced by addition of 0.5 mM IPTG and continue cultivating for 4 h at 26°C. Collected cells were washed with PBS, fixed, incubated for 5 min in PBS with 0.3% Triton X-100 , stained using 10 μg ml-1 DAPI for 30 min at 37°C (Solarbio, China), then washed three times with PBS.
Instrument	Beckman CytoFLEX
Software	FACSDiva for collection and FlowJo (v10) for analysis
Cell population abundance	Populations were validated for purity by a post-sort analysis by FACS
Gating strategy	Every flow cytometry analysis was initiated as follows:<br>FL1/histogram for FITC, PB450H/SSC for DAPI<br>Gating of the Fixble Viability Dye FITC or DAPI negative cells to select positive cells followed by the gating as described in the figures.

- [x] Tick this box to confirm that a figure exemplifying the gating strategy is provided in the Supplementary Information.
